# Successful surgical repair of thoracic outlet syndrome in patient affected by clavicular pseudoarthrosis and chronic sternoclavicular dislocations following traumatic clavicle fracture: a case report

**DOI:** 10.1093/jscr/rjag369

**Published:** 2026-05-16

**Authors:** Matteo Vitali, Filippo Montalbano, Francesca Crippa, Andrea Kahlberg, Roberto Chiesa

**Affiliations:** Department of Orthopaedics and Traumatology, Ospedale San Raffaele, Milano, 20132, Via Olgettina 60, Italy; Università Vita-Salute San Raffaele, Milano, 20132, Via Olgettina 60, Italy; Università Vita-Salute San Raffaele, Milano, 20132, Via Olgettina 60, Italy; Department of Vascular Surgery, Ospedale San Raffaele, Milano, 20132, Via Olgettina 60, Italy; Department of Vascular Surgery, Ospedale San Raffaele, Milano, 20132, Via Olgettina 60, Italy

**Keywords:** TOS, sternoclavicular fracture, reduction and fixation

## Abstract

Thoracic outlet syndrome (TOS) secondary to chronic sternoclavicular (SC) joint pseudoarthrosis is a rare but clinically significant condition. We report the case of a 50-year-old male who developed chronic SC joint instability and neurogenic TOS 2 years after a conservatively treated SC fracture. Preoperative Doppler ultrasound showed dynamic venous outflow impairment. Surgical treatment included clavicular calloclasia, adhesiolysis, anterior scalenectomy, SC joint reduction and fixation, and anchorage stabilization. At 2-month follow-up, the patient achieved complete pain relief, full functional recovery, and resolution of neurological and vascular symptoms. Early surgical management can restore stability and prevent neurovascular complications.

## Introduction

Thoracic outlet syndrome (TOS) is a condition caused by compression of the neurovascular structures passing through the thoracic outlet [1]. It can present after trauma, repetitive overhead activity, or congenital anatomical variations such as a cervical rib or hypertrophied scalene muscles [1]. In recent reviews, 49% of TOS cases reported having a history of single trauma as the cause [1].

Chronic sternoclavicular (SC) joint dislocation is a rare condition accounting for <3% of all shoulder-girdle dislocations, usually resulting from significant trauma to the upper chest or shoulder girdle [2]. If this injury is not properly treated or goes unnoticed, the dislocation can become chronic, characterized by persistent deformity, instability, and sometimes pain associated with arm movement [2]. In chronic cases, the surrounding soft tissue may adapt to the malposition, making delayed reduction difficult or impossible, while also increasing the risk of developing joint pseudoarthrosis [3].

In a patient affected by TOS associated with clavicular pseudoarthrosis and chronic SC joint dislocation, the therapeutic approach must address both the neurovascular compression and the mechanical instability caused by the clavicular displacement, aiming to relieve neurovascular compression while restoring clavicular stability [4].

Initial treatment for chronic SC joint dislocation is typically conservative, focusing on physiotherapy to correct posture, stretch the scalene and pectoralis minor muscles, and strengthen the shoulder stabilizers to reduce mechanical tension across the thoracic outlet [2]. Analgesics, anti-inflammatory drugs, and physical therapies such as heat or ultrasound can help control pain and muscle spasm, while avoiding overhead or repetitive shoulder movements may prevent symptom exacerbation [5].

Meanwhile, treatment for clavicular pseudoarthrosis is typically surgical, since if left untreated, it can lead not only to functional joint limitations and articular pain, but also possible risk of neuro-vascular complications [6].

Surgical management may include thoracic outlet decompression through first rib resection, scalenectomy, or fibrous band release to alleviate neurovascular compression, as well as open reduction and fixation or ligament reconstruction of the sternoclavicular joint to correct deformity and restore stability [6].

Postoperatively, immobilization of the shoulder is followed by progressive rehabilitation to restore motion, muscle strength, and scapulothoracic balance [7]. Long-term follow-up is essential to monitor functional recovery and ensure the absence of recurrence or persistent neurovascular compromise [7].

The patient gave his full agreement to publish his case details and images.

## Case report

We present a rare case of a 50 year old Caucasian clinically depressed male, 170 cm and 60 kg, who reported a polytraumatic event caused by a fall, due to which he sustained a right anterior sternoclavicular dislocation and fracture treated conservatively 2 years prior being accepted at our hospital.

At physical examination, he presented visible deformity of the SC joint and had developed hypermobility of the bony segment with symptoms compatible with clavicular pseudoarthrosis. The patient also lamented chronic pain, limited shoulder mobility, and paraesthesia of the right upper limb, indicative of TOS and joint pseudarthrosis, there was no edema or swelling of the affected limb, but a slight decrease in grip strength was detected compared to the healthy arm.

**Figure 1:**
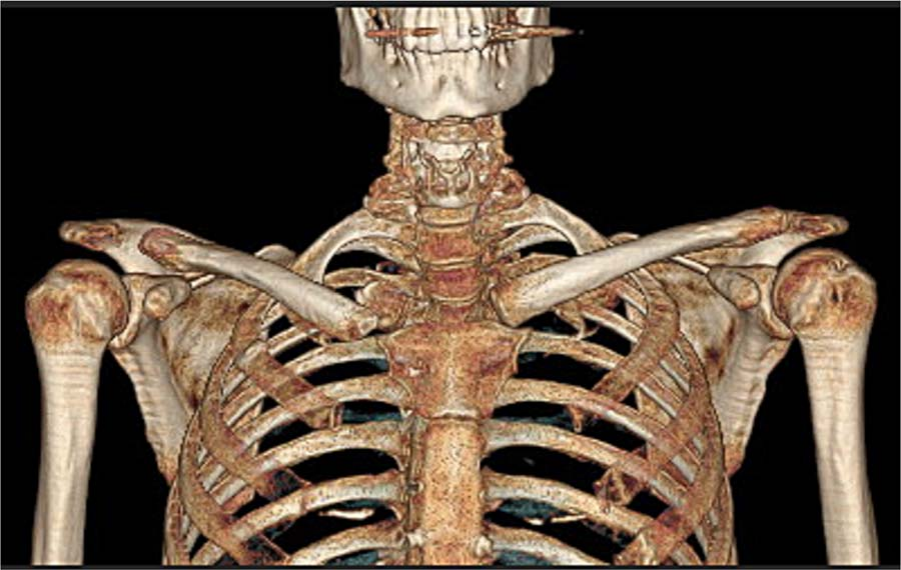
Pre-operative CT imaging showed signs of pseudoarthrosis involving the sternoclavicular joint.

Computed tomography (CT) imaging showed signs of pseudoarthrosis involving the SC joint (Fig. 1).

Preoperative ultrasound Doppler examination found evidence of right subclavian artery moderate atheromasia, with alterations to venous outflow during dynamic manoeuvres on the right side, with risk of developing arterial thrombosis caused by pseudoarthritic fibrous tissue. For these reasons, he was referred to surgical treatment.

**Figure 2:**
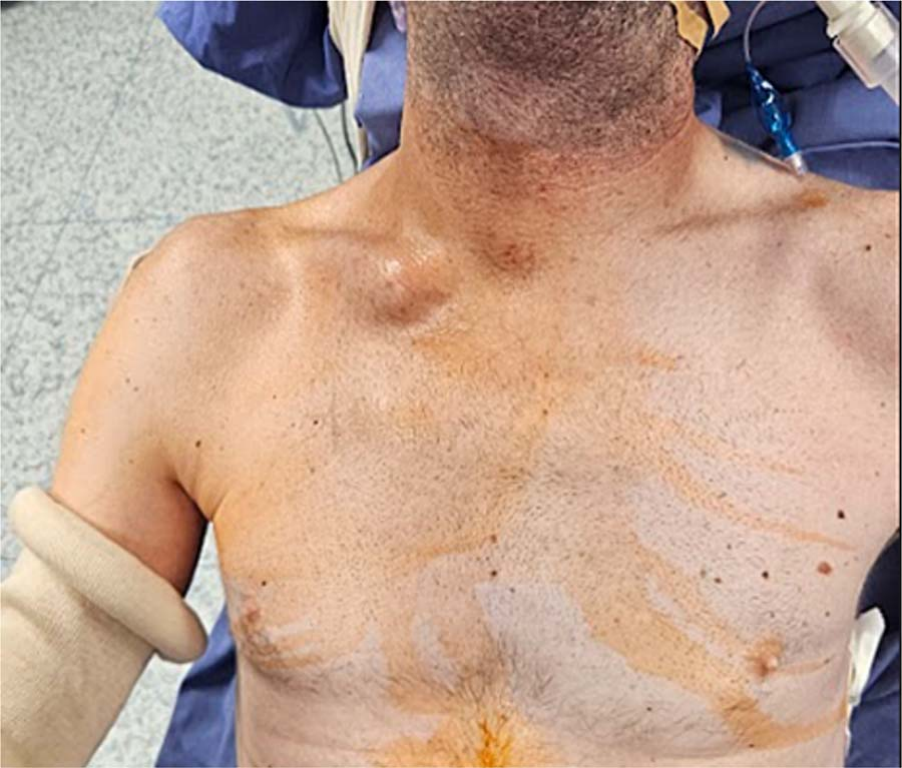
Shows the patient under general anaesthesia, in semi-upright position, before surgery. It also shows evident sternoclavicular joint deformity on the affected side.

Under general anaesthesia, in semi-upright position (Fig. 2), a right supraclavicular incision of about 8 cm was made.

**Figure 3:**
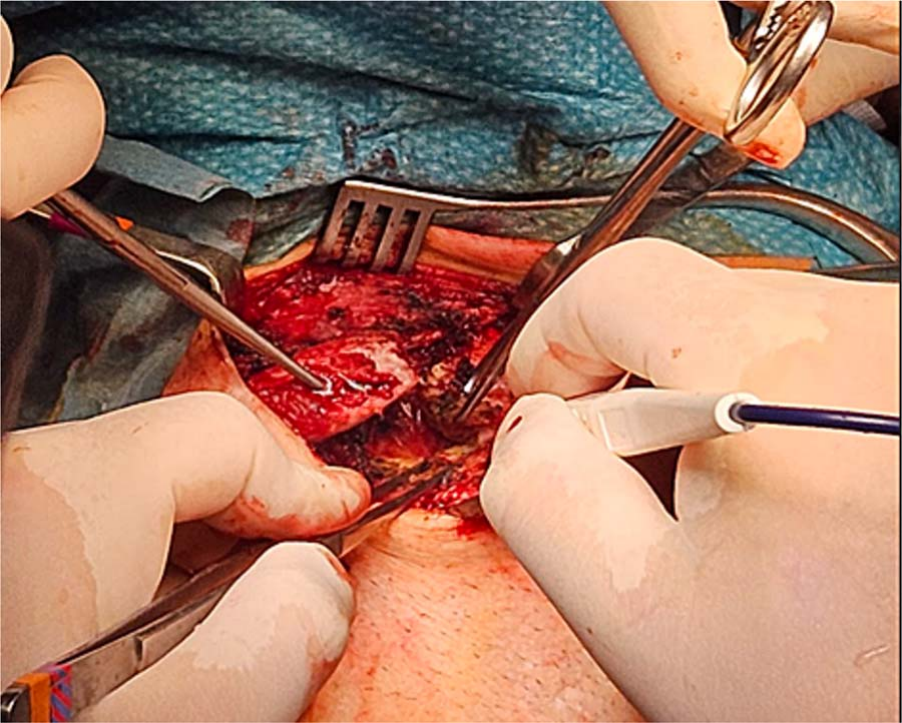
Calloclasia was performed, showing sternoclavicular instability with capsuloligamentous injury. The neurovascular bundle, consisting of the left subclavian artery and the brachial plexus, was isolated. Adhesiolysis was performed.

Through blunt dissection, the bony plane was reached, revealing pseudoarthrosis of the middle third of the right clavicle with abundant callus formation, appearing as the likely outcome of a malunited midshaft fracture. Calloclasia was performed (Fig. 3), showing sternoclavicular instability with capsuloligamentous injury. The neurovascular bundle, consisting of the left subclavian artery and the brachial plexus, was isolated. Adhesiolysis was performed (Fig. 3).

**Figure 4:**
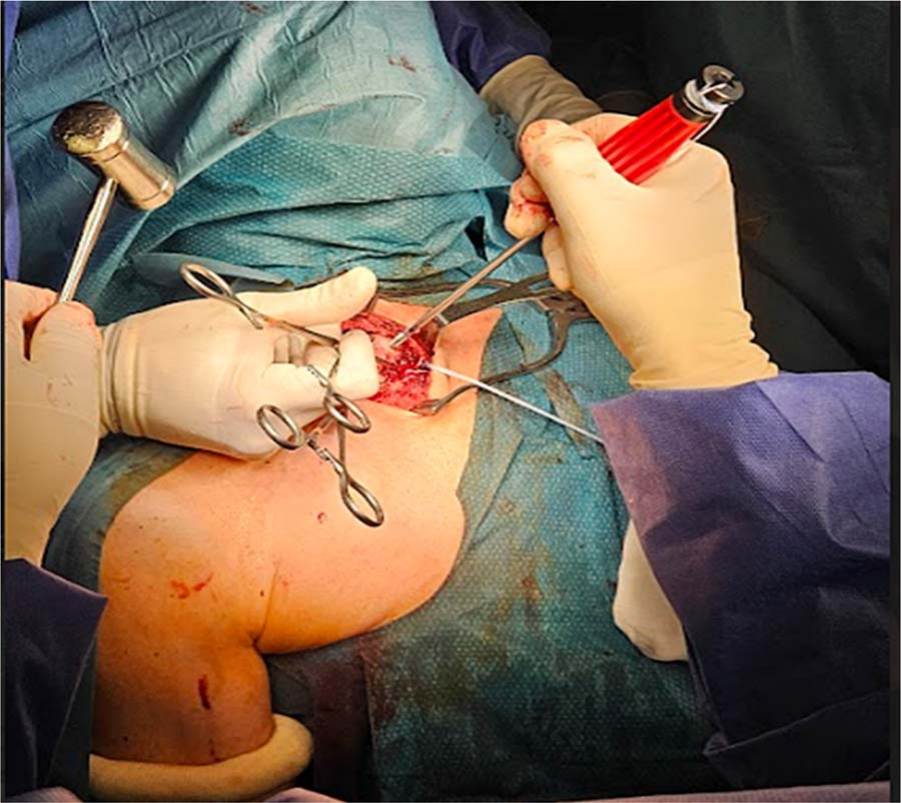
The sternoclavicular joint was reduced and stabilized using anchors and high-resistance sutures.

**Figure 5:**
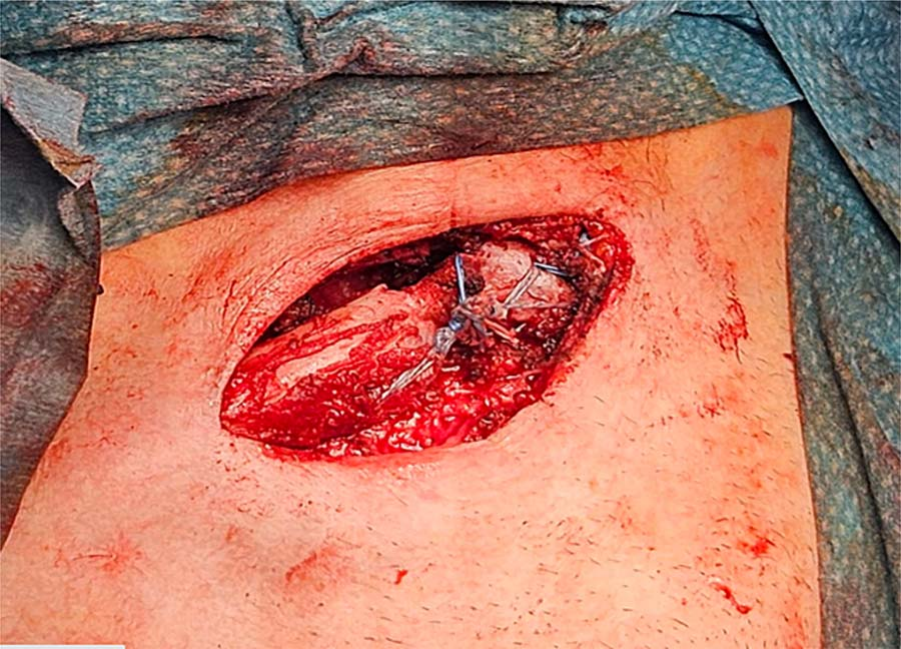
The sternoclavicular joint was reduced and stabilized using anchors and high-resistance sutures.

The right subclavian vein was isolated from fracture stumps, and adhesions were carefully released. The anterior scalene muscle was identified and sectioned. Accurate haemostasis and irrigation were performed. The sternoclavicular joint was reduced and stabilized using anchors and high-resistance sutures (Figs 4 and 5).

**Figure 6:**
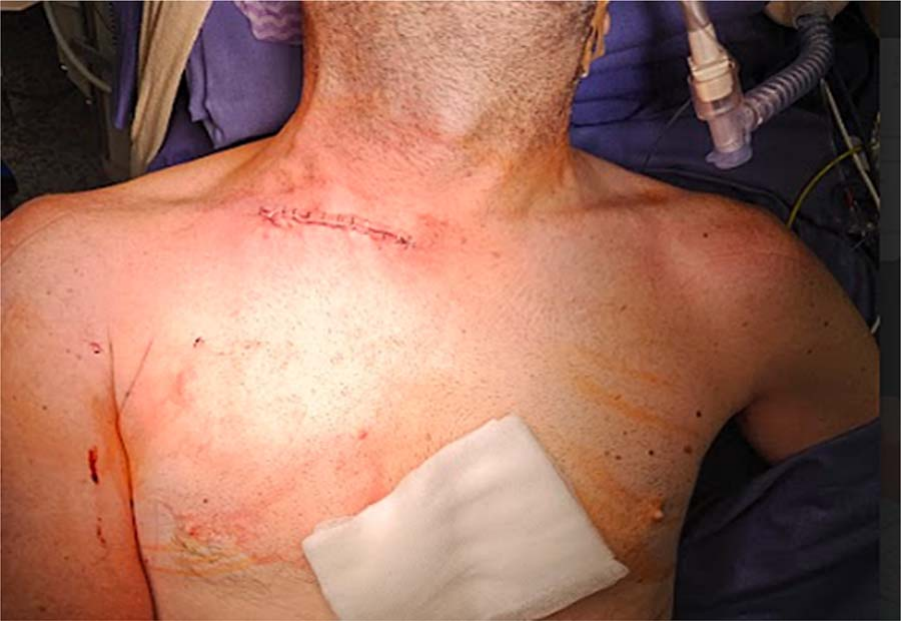
Final surgery result after layered closure.

Final irrigation was performed, followed by layered closure (Fig. 6) and placement of an arm-shoulder immobilizer, which would stay in place for the next 30 days after surgery.

**Figure 7:**
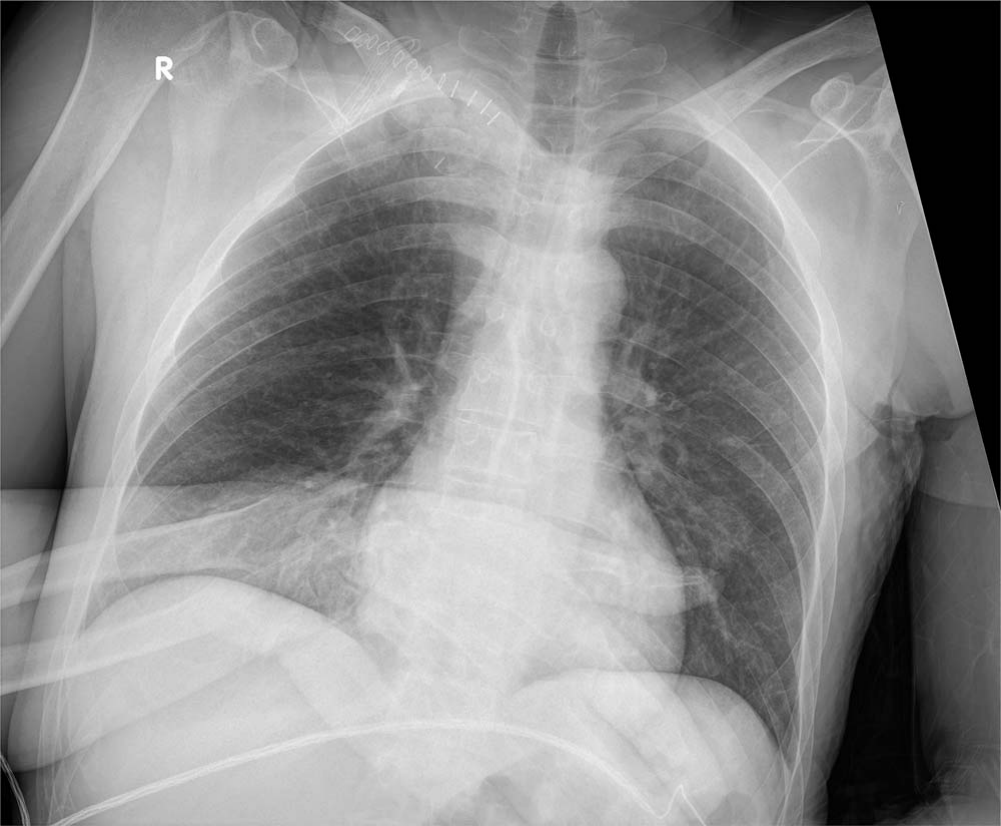
Postoperative X-ray.

Postoperative X-ray imaging documented clear improvement in clavicular alignment compared to preoperative findings, with restoration of anatomical positioning and reduction of the structural abnormality contributing to thoracic outlet compression (Fig. 7).

The patient was then visited 1-month post-surgery, following which he stopped using the arm-shoulder immobilizer and started physical rehabilitation for his right upper limb for the next month. At the 2-month follow-up, we assessed for range of motion and upper limb health, and the patient reported full physical recovery and joint functionality, including improvement in grip strength now comparable to the other arm.

## Discussion

In this presented case, the combined surgical approach involved resection of fibrotic adhesions, anterior scalenectomy, and reconstruction of the SC joint with high-resistance sutures and anchors. This allowed both neural and vascular decompression and re-establishment of mechanical stability. The immediate postoperative course was uneventful, and early rehabilitation contributed to complete functional recovery within 2 months. These results align with previous evidence suggesting that early mobilization following stable fixation can optimize functional outcomes and minimize joint stiffness.

Although surgical management of TOS in patients affected by clavicular pseudoarthrosis or SC joint pathology remains technically demanding due to the proximity of vital structures such as the subclavian vessels and brachial plexus, outcomes are generally favourable when a combination of: preoperative imaging, meticulous dissection, and stable reconstruction are achieved. Long-term follow-up is crucial to monitor for recurrence of neurovascular symptoms or postoperative instability.

This rare case highlights the importance of recognizing post-traumatic deformities as a potential underlying cause of secondary TOS and supports the role of combined decompressive and reconstructive surgery in selected patients with chronic SC dislocation and clavicular pseudoarthrosis with symptomatic neurovascular compression. Early identification and multidisciplinary management, implementing vascular, orthopaedic, and rehabilitation teams, is most important to optimizing long-term function and quality of life.
